# Supplementary material for: Mechanisms governing the pioneering and redistribution capabilities of the non-classical pioneer PU.1
Source: Nat Commun. 2020 Jan 21;11:402. doi: 10.1038/s41467-019-13960-2 (PMC6972792; doi:10.1038/s41467-019-13960-2)
Supplement: Supplementary file 7 — Source data [file 41467_2019_13960_MOESM7_ESM.zip › Source_Data/FigureS2/FigureS2B_MotifScanOutput/KmeansCluster10/homerResults/motif5.similar.html]

motif5

## Information for motif5

C
A
T
G
G
A
T
C
A
G
T
C
C
T
G
A
T
A
G
C
G
A
C
T
C
T
G
A
A
C
T
G
C
T
G
A
A
C
T
G
A
C
T
G
C
A
T
G
G
A
T
C
T
C
G
A
  
Reverse Opposite:  

A
G
C
T
C
A
T
G
G
T
A
C
G
T
A
C
T
G
A
C
A
G
C
T
A
G
T
C
G
A
C
T
C
T
G
A
A
T
C
G
G
A
C
T
A
C
T
G
C
T
A
G
G
T
A
C
  

|  |  |
| --- | --- |
| p-value: | 1e-101 |
| log p-value: | -2.337e+02 |
| Information Content per bp: | 1.721 |
| Number of Target Sequences with motif | 166.0 |
| Percentage of Target Sequences with motif | 4.47% |
| Number of Background Sequences with motif | 136.0 |
| Percentage of Background Sequences with motif | 0.31% |
| Average Position of motif in Targets | 95.5 +/- 52.7bp |
| Average Position of motif in Background | 102.3 +/- 54.8bp |
| Strand Bias (log2 ratio + to - strand density) | 0.2 |
| Multiplicity (# of sites on avg that occur together) | 1.01 |
| Motif File: | file (matrix) reverse opposite |

### Similar de novo motifs found

|  |  |  |  |  |  |  |  |
| --- | --- | --- | --- | --- | --- | --- | --- |
| Rank | Match Score | Redundant Motif | P-value | log P-value | % of Targets | % of Background | Motif file |
| 1 | 0.953 | C A T G G A T C A G T C G T A C A G T C A G T C A G C T C T G A A T C G G C A T A C T G C T A G G A T C | 1e-94 | -217.809958 | 4.26% | 0.31% | motif file (matrix) |
| 2 | 0.956 | G A T C A G T C T C G A T A G C G A C T C G T A A C T G C T G A A C T G A C T G C A T G G T A C | 1e-84 | -194.362647 | 4.01% | 0.33% | motif file (matrix) |
| 3 | 0.927 | A G T C C T G A T A G C G A C T C T G A A T C G C T G A A C T G C T A G C T A G G T A C | 1e-75 | -173.676794 | 5.28% | 0.83% | motif file (matrix) |
| 4 | 0.889 | A G T C C G T A A T G C A G C T C G T A A C T G C T G A A C T G A C T G A C T G | 1e-51 | -118.397950 | 5.23% | 1.26% | motif file (matrix) |
| 5 | 0.760 | A T C G A G T C G A T C G T C A T A G C G T A C G C T A A T C G A C T G C G A T C T A G C T A G | 1e-27 | -62.462071 | 2.59% | 0.59% | motif file (matrix) |
| 6 | 0.759 | A G T C C T G A G T A C A C G T C G T A A C T G C T G A A C T G C T A G | 1e-22 | -50.707148 | 2.88% | 0.87% | motif file (matrix) |
| 7 | 0.752 | A G T C C T G A G T A C A C G T C G T A A C T G C G T A A C T G | 1e-15 | -35.795529 | 4.15% | 1.93% | motif file (matrix) |
